# The biological characteristics and infection dynamics of a novel H3N2 canine influenza virus genotype in beagles

**DOI:** 10.1186/s12985-024-02422-x

**Published:** 2024-07-04

**Authors:** Fei-fei Ge, Hai-xiao Shen, De-quan Yang, Xian-chao Yang, Xin Li, Jian Wang, Shixin Huang

**Affiliations:** Shanghai Animal Disease Control Center, Shanghai, 201103 People’s Republic of China

**Keywords:** Canine influenza virus, 5.1 clade, Biological characteristics, Infection dynamics, Beagles

## Abstract

**Background:**

The canine influenza virus (CIV) outbreak has garnered considerable attention as it poses a significant threat to dog health. During the H3N2 CIV evolution in beagles, the virus formed a new clade after 2019 and gradually became more adaptable to other mammals. Therefore, successfully elucidating the biological characteristics and constructing a canine influenza infection model is required for CIV characterization.

**Methods:**

We performed genetic analyses to examine the biological characteristics and infection dynamics of CIV.

**Results:**

The genotype of our H3N2 CIV strain (from 2019 in Shanghai) belonged to the 5.1 clade, which is now prevalent in China. Using MDCK cells, we investigated viral cytopathic effects. Virus size and morphology were observed using transmission electron microscopy. Beagles were also infected with 10^4^, 10^5^, and 10^6^ 50% egg-infectious doses (EID_50_). When compared with the other groups, the 10^6^ EID_50_ group showed the most obvious clinical symptoms, the highest virus titers, and typical lung pathological changes. Our results suggested that the other two treatments caused mild clinical manifestations and pathological changes. Subsequently, CIV distribution in the 10^6^ EID_50_ group was detected by hematoxylin and eosin (H&E) and immunofluorescence (IF) staining, which indicated that CIV primarily infected the lungs.

**Conclusions:**

The framework established in this study will guide further CIV prevention strategies.

## Background

Canine influenza virus (CIV) belongs to the Orthomyxoviridae family and contains eight single-stranded negative RNA fragments encoding more than 15 viral proteins [1]. Dogs are susceptible to avian influenza virus (H3N2 and H5N1), equine influenza virus (H3N8), or human influenza virus (pdmH1N1 and H3N2) [2–4]. Globally, H3N8 and H3N2 are the major CIV subtypes [4]. H3N8 CIV has been circulating mainly in the United States since 2004 [5], while H3N2 CIV is dominant in South Korea and China [4, 6]. When compared with H3N8 CIV, H3N2 CIV has a wider host range and infects various mammals, including ferrets, guinea pigs, mice, and cats [7–10].

Throughout history, influenza pandemics have been caused by the introduction of new viruses. Pigs are a “mixed container” of influenza A virus (IAV) in birds and mammals, while dogs are the most likely animals to come into contact with humans, and are suspected as potential mixed containers. As one of the most popular pets, dogs have close contact with humans, so CIV spread poses a serious threat to the human influenza virus. However, studies on animals infected with CIV have been conducted. H3N2 CIV pathogenesis is contradictory. Research has shown that H3N2 CIV damages organs and the respiratory tract, with virus titers detected in corresponding tissues [11]. In contrast, low pathogenic H3N2 CIV and even highly pathogenic H5N1 CIV are the main causes of damage to the tonsils and respiratory tract, with virus titers undetected in other organs [12–14]. These results indicate that CIV clinical symptoms and virus replication dynamics are highly dependent on individual differences in the infection strain, the vaccination route, and host immunity [15–18]. Therefore, the comprehensive confirmation of H3N2 CIV pathogenicity in dogs is required. In our study, a new CIV genotype was cultured in MDCK cells supplemented with 2% Dulbecco’s Modified Eagle Medium and trypsin, treated with 1 µg/ml TPCK, and observed under electron microscopy. We also investigated viral infection dynamics in beagles.

## Methods

### Ethics statement

Animal protocols were approved by the Laboratory Animals Ethics Committee of the Shanghai Animal Disease Control Center.

### Phylogenetic analyses

The hemagglutinin (*HA*) genes of six H3N2 CIV isolates (A/canine/China/Shanghai/0103/2019; A/canine/China/Shanghai/0104/2019; A/canine/China/Shanghai/0105/2019; A/canine/China/Shanghai/018/2019; A/canine/China/Shanghai/019/2019; A/canine/China/Shanghai/0114/2019) from Shanghai in 2019 were used for genetic and phylogenetic analyses (Accession numbers; MK758007.1–MK7580054.1). Sequence data were compiled and edited using Lasergene sequence analysis software (Dnastar Inc., Madison, WI, USA). Multiple sequence alignments were conducted using CLUSTAL W. The maximum likelihood tree for origin analysis was constructed in MEGA 4 using 1,000 bootstrap replications [19, 20].

### Virus propagation and electron microscopy

A/canine/Shanghai/0103/2019 (H3N2) was isolated from a stray dog in Shanghai, China. The virus was propagated in 9 day old specific pathogen-free (SPF) embryonated chicken eggs at 37 °C for 48 h and stored at -80 °C. Viral titers were evaluated using the 50% egg-infectious dose (EID_50_/ml) method and calculated using the Reed–Muench method [21]. For virus isolation using MDCK cells, 1 µg/ml TPCK-treated trypsin was used to support viral growth. Infected cells were incubated under 5% CO_2_ at 37 °C for 72 h. Supernatants were harvested and further passaged. Then, infection was detected using RT-PCR (Qiagen, Shenzhen). The H3N2 virus (A/canine/Shanghai/0103/2019) was adsorbed onto a carbon parlodion-coated copper grid for 2 min. Excess suspension was removed by blotting with filter paper, and the grid was immediately stained with 1% phosphotungstic acid for 10 min. Excess stain was removed using filter paper, after which samples were examined using a transmission electron microscope (Hitachi, Japan).

### Clinical studies and viral challenge

Sixteen beagles (10 weeks old) were obtained from the Experimental Animal Center (Runde Biotechnology Co., Ltd., Shanghai, China). Prior to studies, serum samples were collected from all dogs and subjected to hemagglutination inhibition (HI) assays to ensure that animals had not been exposed to H3N2 CIV. Four dogs were used as controls. For intranasal administration, the 12 remaining dogs were divided into three groups and intranasally inoculated with 1 ml of 10^4^, 10^5^, or 10^6^ EID_50_ of A/canine/Shanghai/0103/2019. The control group was intranasally inoculated with the same volume of sterile phosphate-buffered saline (PBS). Dogs were housed in separate cages and observed for 10 days after infection.

Nasal swabs were collected, during which time clinical symptoms were monitored. The clinical score of each dog was evaluated using a previously described scoring system [22]. Nasal secretions were collected from left and right nostrils every day until day 10 after inoculation (0–10 days post infection (dpi)) and diluted in 1 ml of PBS plus 1% penicillin and streptomycin. Nasal swabs were used for measuring EID_50_ values using 9–11 day old embryonic chicken eggs. Two dogs from the control group and two from each experimental group were then humanely euthanized at 5 dpi. Turbinates, tracheas, and lung tissues were collected, and EID_50_ sample values determined. Briefly, 1 ml of sterile PBS was added per 1 g of collected tissue, which was then ground in a liquid nitrogen homogenizer. Supernatants were collected by centrifugation and inoculated at different dilutions into 9 day old chicken embryos. The two remaining dogs in groups were monitored until day 10.

### Serological testing

Blood samples were collected from dogs at 2, 4, 6, 8, and 10 dpi. Approximately 500 µl of serum was collected from each dog and stored at -20 °C until required. One volume of serum was mixed with three volumes of receptor-destroying enzyme (RDE, Denka Seiken Co., Ltd.), incubated for 18 h at 37 °C and then 30 min at 56 °C. Antiserum titers were determined using HI assays. The 1% red blood cells used in this study were collected from SPF cocks and diluted in sterile PBS.

### Histopathological examinations using hematoxylin & eosin (H&E) and immunofluorescence (IF) staining

Liver, spleen, lung, trachea, and intestinal tissues collected at 5 dpi were fixed in 10% formalin for > 48 h. Samples were washed overnight, dehydrated in alcohol, and embedded in paraffin. Next, paraffin-embedded tissues were cut into 4–7 μm thick sections and deposited on glass slides. Sections were then mounted and left overnight at 37 °C prior to H&E staining.

IF staining was performed on deparaffinized and rehydrated tissue sections. Briefly, sections were first treated with antigen unmasking solution (Vector Laboratories, California, USA) in a pressure cooker. After blocking in 0.1% Sudan black B for 15 min and 1% bovine serum albumin/PBS at room temperature for 30 min, membranes were incubated with a primary antibody against IAV nucleoprotein (Abcam) at 4 °C overnight. This was followed by incubation with Cy5-conjugated goat anti-mouse IgG (Abcam) for 30 min, after which sections were stained with 4’,6-diamidino-2-phenylindole (Thermo Fisher Scientific). All sections were examined and images captured using a Carl Zeiss LSM780 confocal microscope.

### Statistical analyses

Statistical significance was determined using one-way analysis of variance with *post hoc* Tukey’s multiple-comparison tests using GraphPad Prism (v.7.02) software (GraphPad Software, Inc., La Jolla, CA, USA). P values < 0.05 indicated statistical significance.

## Results

### Phylogenetic analysis

**Fig. 1:**
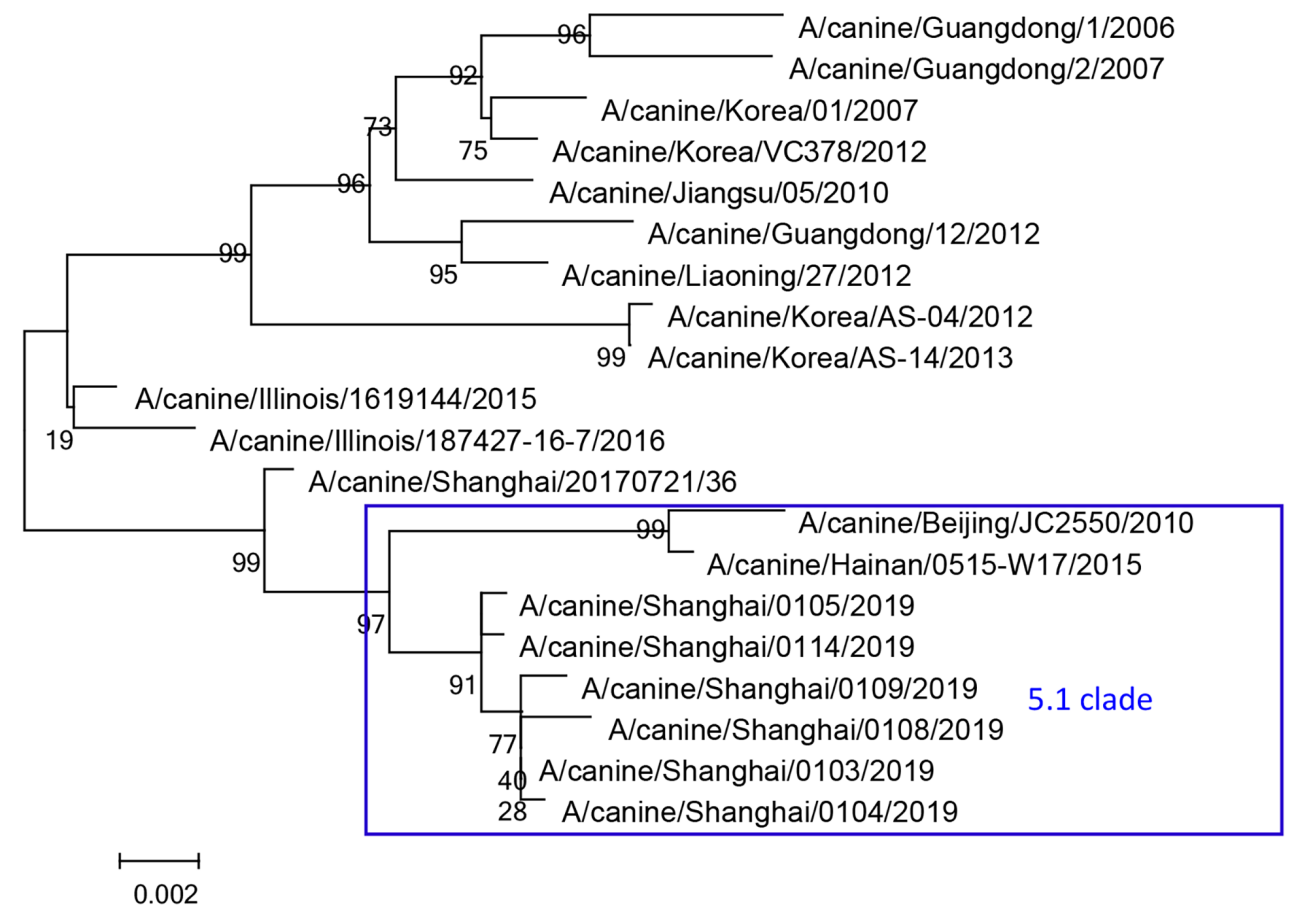
A phylogenetic tree showing the hemagglutinin segment of H3N2 CIVs. The 2019 isolates included in this study are highlighted in the blue square

To better characterize genetic variations in the six isolated H3N2 CIVs from Shanghai, the *HA* segment of all six H3N2 CIVs were analyzed and fell into clade 5.1, which is prevalent in China (Fig. 1). Our six isolates belonged to the same clade as the reference strains [23].

### Virus propagation and electron microscopy

**Fig. 2:**
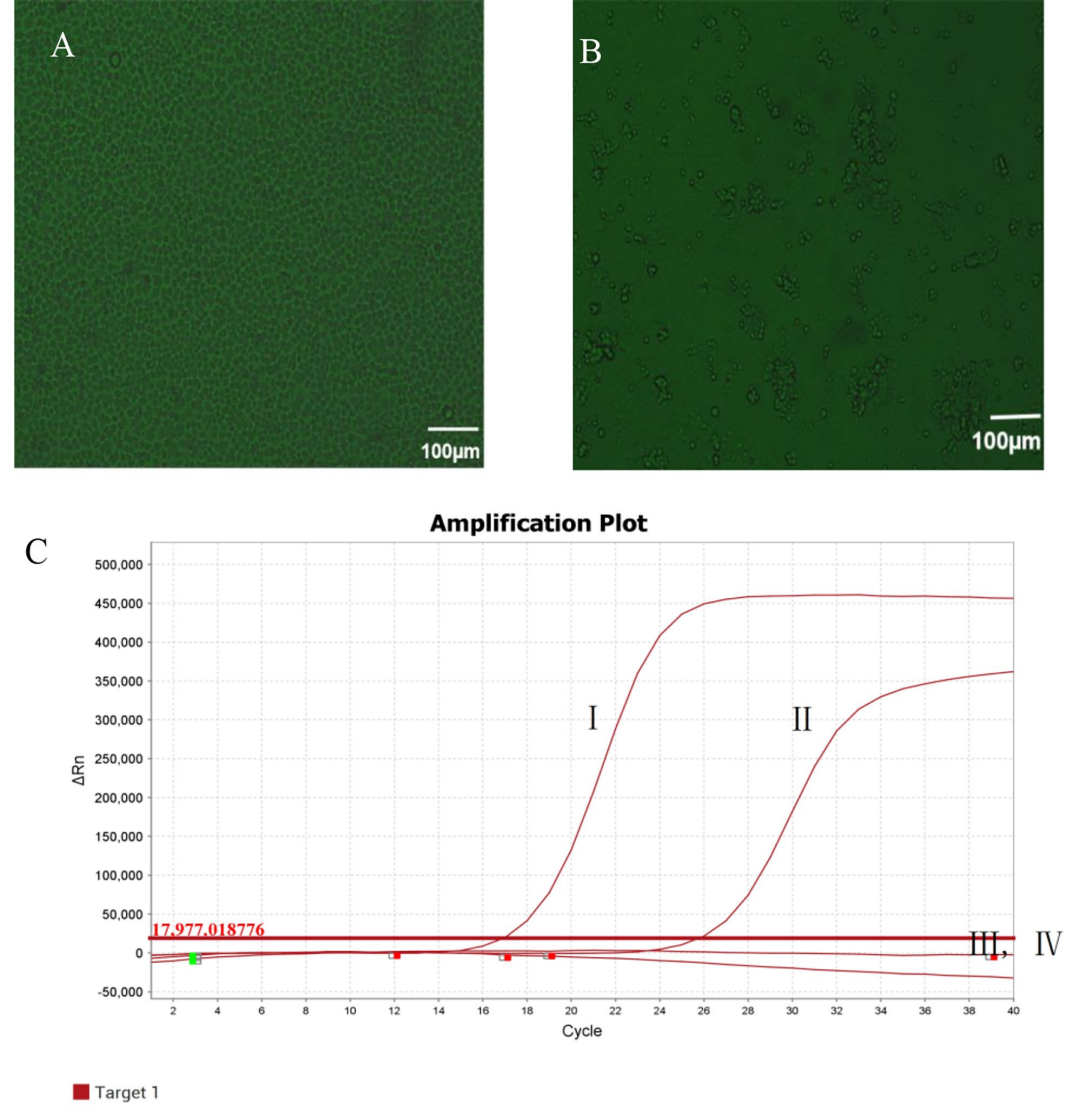
CIV cytopathic effects on MDCK cells at 36 h post-inoculation (hpi) (100× magnification). **(A)** Mock-inoculated MDCK cell culture (normal cells). **(B)** A/Canine/Shanghai/0103/2019-inoculated MDCK cells showing rounded and clustered cells. **(C)** Amplification Plots I: A/Canine/ Shanghai/0103/2019-inoculated MDCK cells; II: Positive control; III: Mock-inoculated MDCK cells; and IV: Negative control

**Fig. 3:**
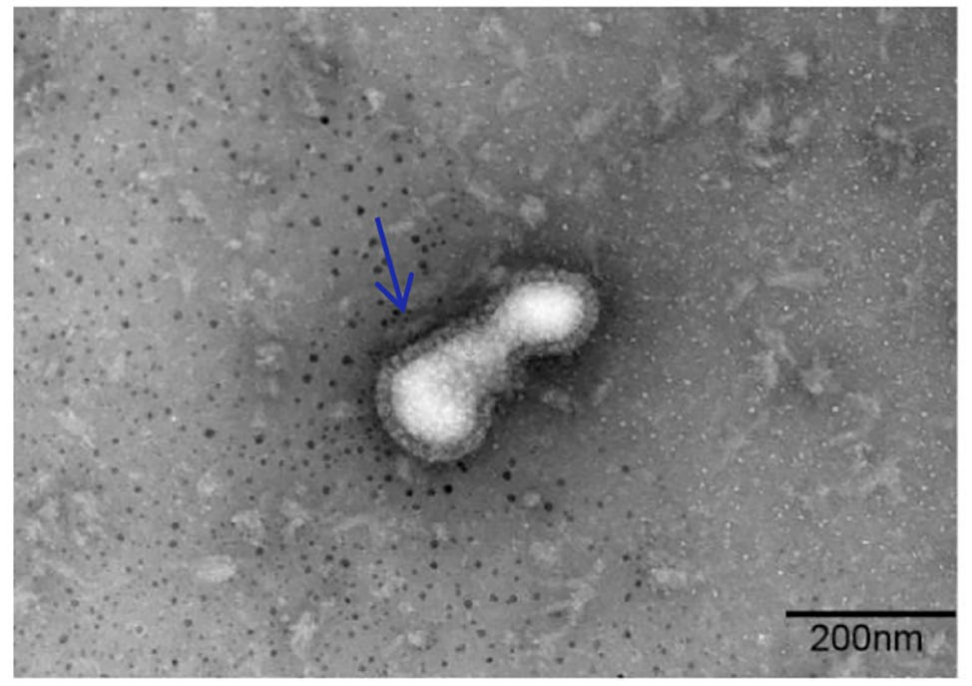
Negative electron microscopy staining of H3N2 CIV (A/canine/Shanghai/0103/2019) (blue arrow)

To further investigate viral biological characteristics, MDCK cells were used. The isolate showed remarkable cytopathic effects toward MDCK cells (Fig. 2A and B). H3N2 CIV was detected using RT-PCR (Qiagen, Shenzhen) (Fig. 2C). Virus size and morphology were both examined using transmission electron microscopy; the average size was 100 nm, and spikes were observed on spherical surfaces (Fig. 3).

### Clinical symptoms

**Fig. 4:**
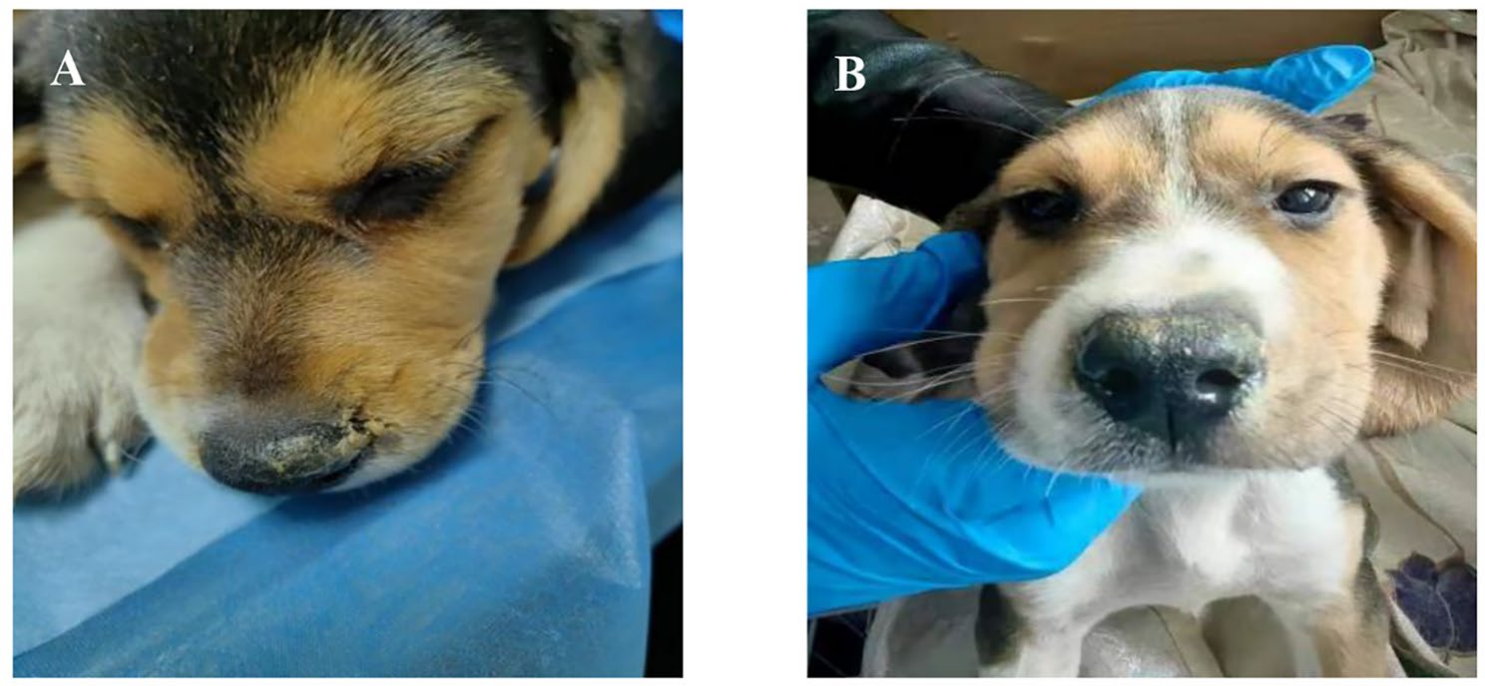
Infected dogs show depression **(A)** and a streaming nose **(B)** at 2 dpi

Different CIV dose replication and pathogenicity outcomes were systematically compared in beagles. All challenged dogs showed respiratory symptoms at 2 dpi, including sneezing, nose clearing, depression, and coughing, which continued until day 8 (Fig. 4A, B).

**Fig. 5:**
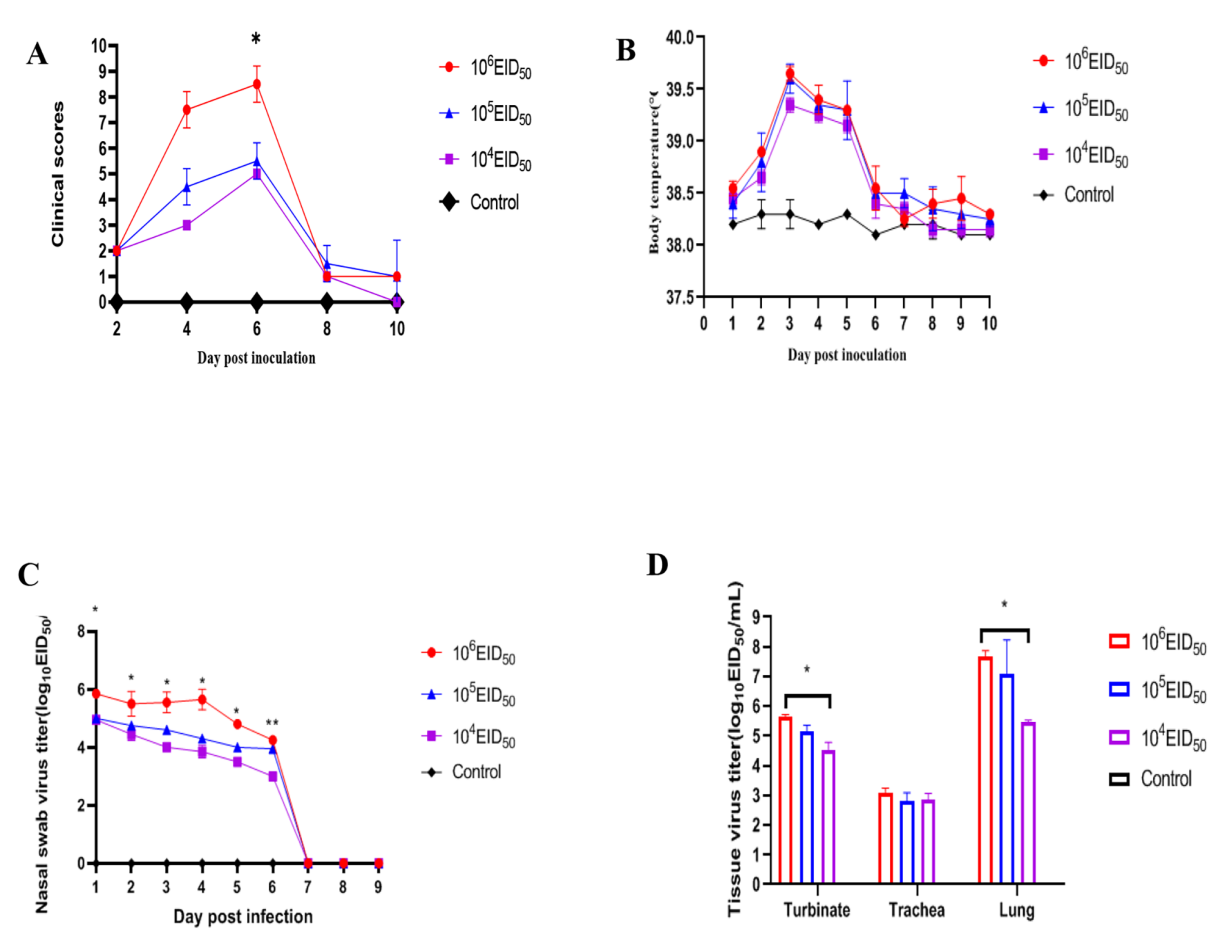
Dogs were inoculated with 10^6^, 10^5^, or 10^4^ EID_50_ (*n* = 2) of the A/canine/Shanghai/0103/2019 virus or PBS (*n* = 2) as a control. Nasal turbinate, trachea, and lung tissues were collected after humanely euthanizing two dogs in each group at 5 dpi. **(A)** After infection with H3N2 CIV at days 2, 4, 6, 8, and 10, clinical scores were recorded; **(B)** Body temperatures were recorded at 1–10 dpi; **(C)** Nasal swabs were evaluated by EID_50_ assays at 1–9 dpi (note: no significant difference between 10^6^ EID_50_ and 10^5^ EID_50_ groups at 6 dpi were recorded; a significant difference was recorded between 10^6^ EID_50_, 10^5^ EID_50_, and 10^4^ EID_50_ groups at 6 dpi). **(D)** Tissue virus titer. EID_50_ assays were conducted to evaluate virus titers in turbinates, tracheas, and lung tissues at 5 dpi (*P < 0.05)

When the infectious dose was 10^6^ EID_50_, clinical symptom scores were greater than the other two experimental groups at 4 and 6 dpi. The difference between the 10^6^ EID_50_ group and the other groups was significant at 6 dpi (*P* < 0.05) (Fig. 5A). When compared with the control group, body temperatures in infected dogs fluctuated to varying degrees, and fever always occurred at 2–6 dpi (Fig. 5B).

The virus was detected within 24 h post-inoculation, and virus shedding lasted for 1–6 days in all challenged dogs. The virus titer in the 10^6^ EID_50_ group was significantly greater than in the other two groups at 1–5 dpi (*P* < 0.05). No virus was detected on the 7th day, showing that virus shedding terminated at 7 dpi in beagles (Fig. 5C). As shown (Fig. 5D), virus titers were evaluated in turbinates, tracheas, and lungs in infected dogs at 5 dpi. In turbinates and lungs, but not in tracheas, virus titers in 10^6^ EID_50_ and 10^5^ EID_50_ groups were significantly greater than titers in the 10^4^ EID_50_ group (*P* < 0.05).

### Seroconversion (HI titers)

**Fig. 6:**
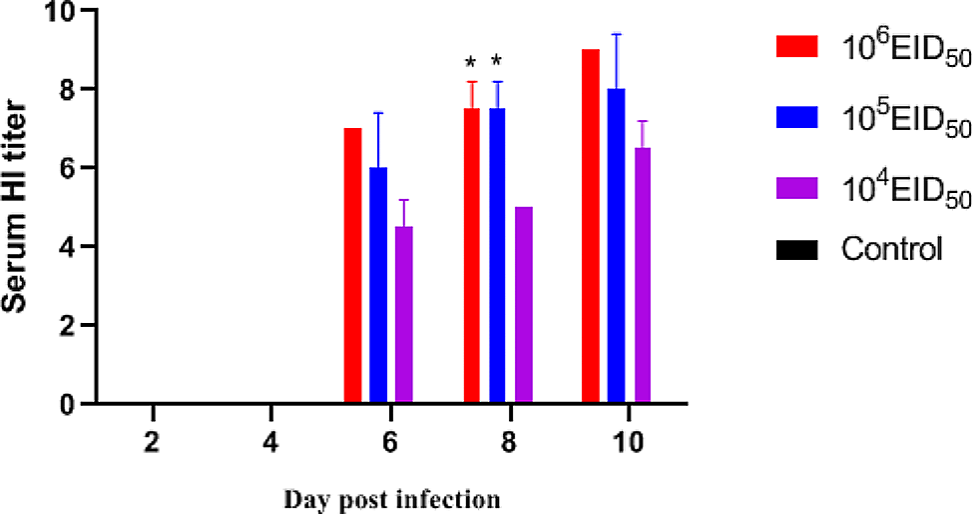
Hemagglutination inhibition (HI) titers showing serum-specific antibody levels (*n* = 2) (*P < 0.05)

To determine antibody responses to CIV-H3N2, challenged dogs were analyzed and were seropositive at 6–10 dpi. At 6 dpi, serum antibody levels in all challenged groups were increased. When compared with 10^4^ EID50 group levels, 10^5^ EID_50_ and 10^6^ EID_50_ group levels were significantly higher at 8 dpi (*P* < 0.05) (Fig. 6).

### Anatomical examinations

**Fig. 7:**
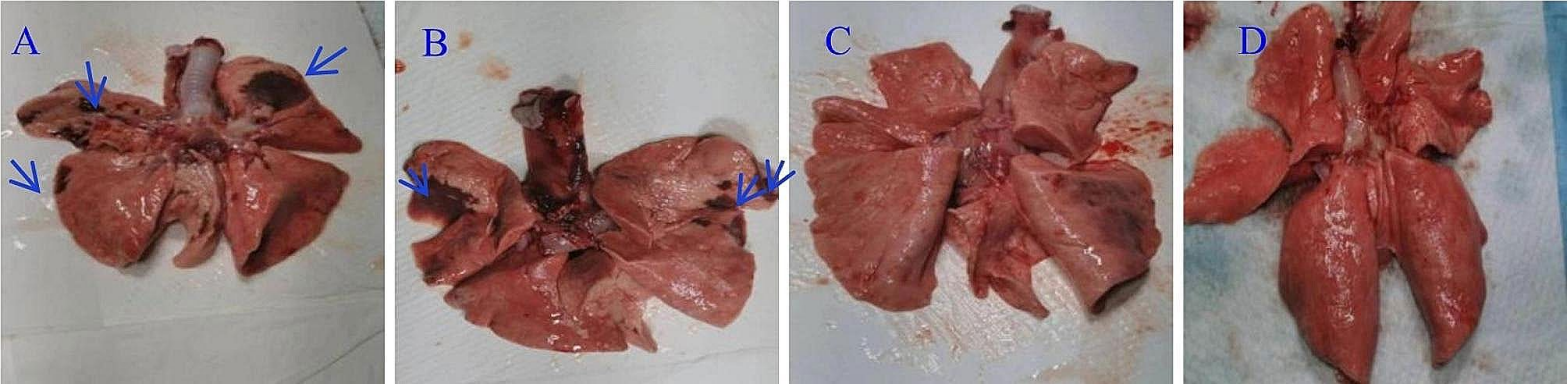
Macroscopic images showing beagle lungs. **(A)** 10^6^ EID_50_; **(B)** 10^5^ EID_50_; **(C)** and 10^4^ EID_50_ challenge groups, **(D)** normal beagle lungs. The blue arrows indicate tissue damage

Influenza can cause acute lung injury. An anatomical lung examination revealed that 10^6^ EID_50_ and 10^5^ EID_50_ groups showed obvious lesions; lung surfaces had visible bleeding spots. However, visible lesions were barely detected in 10^4^ EID_50_ group lungs (Fig. 7A–D).

### H&E and IF staining

**Fig. 8:**
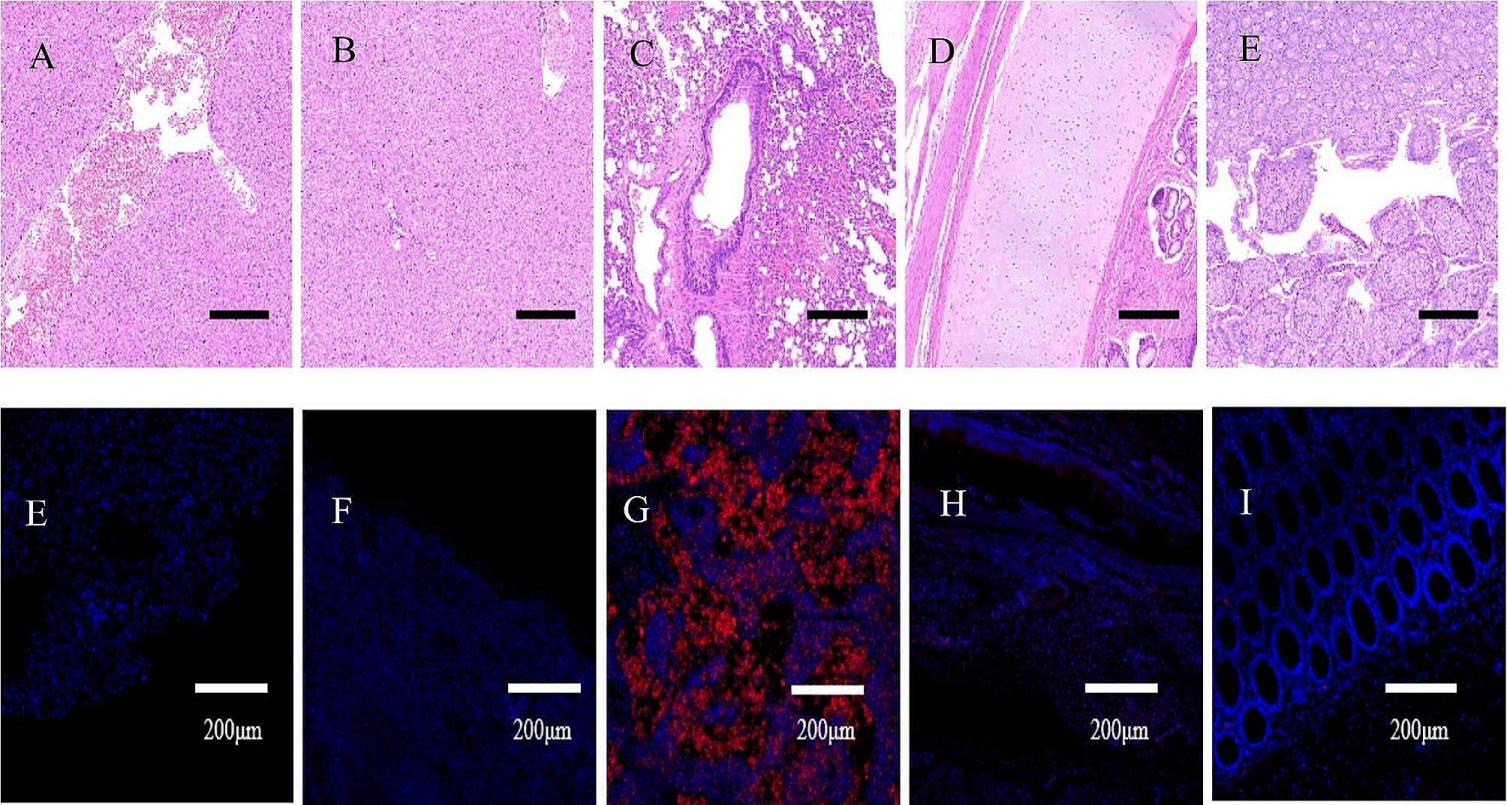
At 5 dpi, liver, spleen, lung, trachea, and intestinal tissues were collected for hematoxylin and eosin **(A–D)** and immunofluorescence staining **(E–H)**

To identify virus-infected tissues, at 5 dpi, liver, spleen, lung, trachea, and intestinal tissues were collected from the 10^6^ EID50 group for H&E and IF staining. Lung pathological lesions, including interstitial pneumonia and hepatocyte degeneration and swelling, were observed (Fig. 8A). As shown (Fig. 8B), strong CIV antigen staining was observed in lungs, while no CIV antigen staining was detected in the liver (Fig. 8).

## Discussion

As a popular companion, dogs typically have close contact with humans and are considered potential intermediate IAV hosts [24]. Canine influenza has occurred in Shanghai and other places in recent years, and potentially threatens public health safety. Research on H3N2 subtype CIV pathogenicity can help us better understand disease pathogenesis, vaccine research and evaluation. Therefore, six H3N2 CIV strains from Shanghai were analyzed according to the report by Chen et al.; these strains belonged to the 5.1 clade, which is now prevalent in China [23]. We also confirmed that H3N2 subtype CIV caused high fever, shortness of breath, cough, and severe lung consolidation in dogs, largely consistent with published H3N2 CIV pathogenicity [25, 26]. To prevent further CIV, infection dynamics in beagle were investigated. We compared clinical scores, body temperatures, nasal swab wash virus titers, tissue virus titers, and lung tissue damage between different titer groups (10^4^ EID_50_, 10^5^ EID_50_, and 10^6^ EID_50_). Overall, when compared with other lower intranasal doses, the intranasal 10^6^ EID_50_ dose caused obvious clinical symptoms and tissue damage. At 6 dpi, more than 2^4^ HI titers were induced in challenged groups. The body induces immune responses against IAVs, which often cause secondary bacterial diseases and lung diseases after infection [27].

Influenza virus mainly infects the upper respiratory tract and bronchial epithelial cells, but in severe cases, it can spread to the bronchioles and alveoli to cause interstitial pneumonia. To assess the degree of lung injury caused by different challenges, we collected lung tissues from infected beagles at 5 dpi. With increasing dose, clinical symptoms in dogs were more obvious; lung lesions were more serious, and the attack dose positively correlated with disease severity. Furthermore, we tested the tissue tropism of the virus in liver, spleen, lung, trachea, and intestine samples. H&E staining revealed that hepatocytes were swollen and degenerated, and lungs showed interstitial pneumonia. Using IF, CIV mainly infected the lungs but not the liver, spleen, trachea, or intestines. From our analyses, the clinical symptom observation and body temperature detection have the error of human factors, and the two can be used as the reference for the animal test of H3N2 subtype CIV infection. Severe lung consolidation occurred in 10^6^ EID_50_-infected dogs. Therefore, the degree of consolidation in lungs was an important criterion for the incidence of canine. Hence, our experiment lays a solid foundation for studying CIV pathogenesis and evaluating vaccine efficacy.

IAV pandemics are caused by novel viruses that efficiently sustain transmission into human populations with limited herd immunity. Dogs are potential mixing vessels for avian and mammalian IAVs and represent a human health concern due to their susceptibility to infection, their numbers, and close physical contact with humans [28]. It was reported that our isolates belonged to a distinct branch with mutations related to mammalian adaptation [29]. Our study provides increasing evidence that canine population surveillance for IAVs is an important component of pandemic preparedness.

## Data Availability

No datasets were generated or analysed during the current study.
